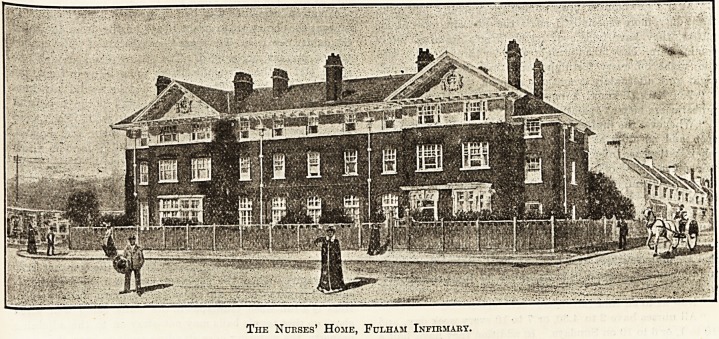# The Hospital. Nursing Section

**Published:** 1905-07-15

**Authors:** 


### The Hospital.

### Nursing Section.

Contributions for this Section of "The Hospital" should be addressed to the Editor, "The Hospital" Nursing Section, 28 & 29 Southampton Street, Strand, London, W.C.

No. 981.—Vol. XXXVIII. SATURDAY, JULY 15, 1905.

### Notes on News from the Nursing World.

### THE QUEEN AND THE ARMY NURSES.

The feature of the annual dinner, last week, of Queen Alexandra's Imperial Military Nursing Service was the reading by each member of a telegram from the Queen. The telegram was addressed to Lady Downe, who presided, and was as follows :—"Will you give the nurses who dine with you to-night my best wishes, and thank them for all they have done in furtherance of a scheme which is so near to my heart.—Alexandra." A telegram of thanks, expressing deep appreciation of Her Majesty's kindness, was sent in reply. A letter was also read by Miss Browne, the matron-in-chief, from Countess Roberts, who expressed her deep regret and disappointment at her inability to be present at the dinner. The guests were received by Miss Browne, R.R.C., Miss Becker, R.R.C., Miss Monk and Miss Cave of the Nursing Board.

### RETIREMENT OF MISS SIDNEY BROWNE, R.R.C.

It will be learnt with deep regret that Miss Sidney Browne will not long continue to hold the position of matron-in-chief of Queen Alexandra's Imperial Military Nursing Service. Rumours to this effect were last week confirmed by Lady Downe, who, in the capacity of president of the dinner of members of the service, intimated that Miss Browne would not be present at another gathering as matron-in-chief. The temporary appointment of Miss Browne was first announced by us in April 1902, and a few months later it was officially intimated that the appointment had been made permanent. Miss Browne entered the Army Nursing Service at Netley on July 1st, 1883. She served in the Nile Campaign in 1885, receiving the medal and bronze star in recognition of her services. Subsequently she was sister at the Herbert Hospital, Woolwich, superintendent sister at the Military Hospital, Malta, and at the Connaught Hospital, Aldershot. During the South African war she was superintendent of No. 3 General Hospital, and also of No. 12, and she distinguished herself in such a marked manner that she was awarded both the medal and the decoration of the Royal Red Cross. As matron-in-chief of Queen Alexandra's Imperial Military Nursing Service, Miss Browne has not only won golden opinions owing to her tact, ability, and the kindness which she has shown to all her subordinates, but she has done much to render the service efficient and popular. In choosing her successor, we trust that the authorities will be careful to make an appointment of a similar character, and avoid the serious mistake of selecting either a narrow-minded martinet or a person whose idea of duty is to withhold information to which the public are entitled.

### HONOURS FOR MISS LÜCKES.

A well deserved compliment has been paid to Miss Lückes, the matron of the London Hospital, by the members of the medical and surgical staff. In recognition of the completion of the 25th year of her matronship, and as a memento of the staff's appreciation of the great work performed by her in creating the nursing school of the London Hospital, and in raising the standard of nursing to its present high state of efficiency, they have presented her with a silver tea and coffee service, suitably inscribed. It is seldom that the matron of a great institution remains at her post for a quarter of a century, and we congratulate Miss Lückes upon the length of her term of office as well as upon the signal token she has just received that her services continue to be appreciated.

### THE CAREER OF MISS PETER.

The retirement of Miss Peter from the office of general superintendent of Queen Victoria's Jubilee Institute, which will take effect at the end of September, will bring to a close a most interesting and useful career. It is just thirty years ago since Miss Peter entered the Royal Infirmary, Edinburgh, as a probationer. At the conclusion of her training she became charge nurse in the old building, and then, when the present building was finished, night superintendent. Subsequently, for eight years and a half, she was superintendent of the Children's Hospital, Edinburgh. In November 1888, Miss Peter was engaged as superintendent of the Scottish Branch of Queen Victoria's Jubilee Institute, and came from Scotland to the Bloomsbury Square and Battersea Homes with the view of obtaining an insight into district work. She began work in Scotland in April 1889, and commenced her duties with two nurses in rooms in North Charlotte Street, Edinburgh. In September 1892 she was asked to act as general inspector for the Institute. By that time, however, there had been a great development in Scotland, and Miss Peter had under her in training at a large house in Castle Terrace, Edinburgh, from eighteen to twenty probationers, as well as the oversight of many branches in Scotland. The first general inspector for the institute was Miss Paget, and the second Miss Mansell, subsequently Mrs. Cheadle, and a few years after Miss Peter's appointment the title was changed to that of general superintendent, because the work had grown so much that it was essential to have several inspectors for England in addition to Scottish and Welsh superintendent and inspectors. The headquarters of the Institute were removed in July 1903 from St. Katharine's Precincts, Regent's Park, to

120 Victoria Street, S.W. Miss Peter has seen the number of associations formed or affiliated to the Institute reach 701, while the number of Queen's nurses on the roll on July 1 was 1,220. That the movement is still making steady progress may be gathered by the fact that the applications for affiliation in the last half-year were 15, as against nine the corresponding six months of 1904. From the first Miss Peter has steadily exercised her influence in order to maintain a high standard of nursing and an adequate remuneration for district nurses. She has for years been a member of the Royal National Pension Fund for Nurses, and is a very staunch advocate of its objects. Miss Peter has for some time acted as nurses' representative on the committee of the Junius S. Morgan Benevolent Fund, where her experience and counsels have been much appreciated. At the end of 30 years it is only due to her to affirm that her labours have been widely and warmly appreciated in the nursing world.

### EXCHANGE OF HOSPITAL NURSES.

A conference took place at the County Hall, Wakefield, last week, respecting the proposed establishment amongst the authorities of the isolation hospitals of the West Riding of Yorkshire of a scheme for the temporary transfer or loan of nurses between hospitals to meet fluctuating requirements. Within the administrative county there are nearly 40 isolation hospitals for general infectious diseases and 54 for small-pox alone. The nurses attached to these institutions are about 300, and under the existing system there are occasions when particular hospitals are overstaffed owing to the absence or small number of patients, whilst, on the other hand, in cases when an unusual number of admissions occur there is a difficulty even in obtaining the services of special nurses at high salaries. At the instance of the chairman, the position was explained by Dr. King at the conference, and he declared his conviction that the scheme of exchange would tend both to economy and efficiency. Eventually, after many of the delegates had expressed themselves in favour of the proposal, a committee was appointed to arrange details, which will be laid before another conference, to be held in due course. It would certainly be a mistake to adopt in haste a scheme which might be repented of at leisure. We are afraid, for instance, that when emergency nurses were asked for there would be a disposition to send the least rather than the most experienced, with the result that the sick would suffer from the arrangement. Moreover, we fancy that many fully qualified nurses would not care about being "exchanged" in the manner suggested, and never quite knowing where they would remain for any length of time. On the other hand, there are some who are glad to have all the experience they can get, and the exchange system might afterwards prove useful to them in private nursing by increasing their adaptability.

### AN IRISH CORONER ON TRAINED NURSES.

It transpired in evidence at an inquest held last week in the Sheil Hospital, Ballyshannon, on a patient who died five days after an operation, that no fully-trained nurse is in attendance during the day. This appears to us the most important fact which was brought out in the course of a long inquiry rendered necessary by the statement of Dr. Creighton, one of the visiting surgeons of the Sheil Hospital, that the death of the patient was due to contamination of the dressings, which he attributed to want of professional knowledge. The jury, it is true, found that no blame attached to the nursing staff in the matter, but even assuming that every possible precaution were taken, and that Dr. Creighton was under an erroneous impression, we think that the admitted absence of a trained nurse during the day justifies apprehension lest mistakes should be made. The coroner, we observe, defended the existing state of affairs, and said that "they were sick, sore, and tired of the Local Government Board and trained nurses." If the jury shared this opinion, we are not surprised that they were satisfied that "everything possible had been done for the deceased."

### UNFOUNDED CHARGES AGAINST CHESTER NURSES.

A considerable amount of time was occupied at the last meeting of the Chester Guardians in discussing complaints which had been made against the workhouse nurses by the master and matron. At the outset it was proposed not to go into the matter in the presence of representatives of the press, on the ground that if the story reached the public it might create a scandal. But the majority of the Guardians rightly held that there would be a greater scandal if they tried to hush the matter up. However, the complaints of the master and matron, which were to the effect that night nursing was a farce, that an old woman was badly injured during the absence of nurses from the ward, that other patients were kept in bed and refused afternoon tea as a punishment, and that the feeble sick were not properly fed, all fell to the ground on examination. They were called before the Guardians to set them forth, and confronted with the head nurse, who denied that the charges were true. She was supported by witnesses, whose evidence was of such a convincing character, that at the close it was unanimously decided not to proceed with the matter further. One Guardian said that having heard the doctor, the matron, and the nurse, he considered that the charges were perfectly frivolous. The report of the case bears out this view, and it also raises the question whether the authors are suitable persons for the posts they occupy.

### NURSING THE SCHOOLBOY.

The final instalment of an extremely interesting contribution on "Nursing the Schoolboy" by the matron of Denstone College Sanatorium appears in another column. Those who have read it will like to know that it was suggested to the author by her own experience when she needed outside help, and also by her conviction that the schoolboy is really little understood even by matrons of many years' standing. There is another point of importance which influenced her. As mothers do not see much of their boys after they go to school, she thinks that this loss, which means a great deal in the formation of character, may to some extent be supplied by the influence of a matron with whom they are brought closely into contact.

Of course, the primary function of the matron of a college sanatorium is to nurse the boys efficiently when they are ill, but there can be no doubt that in the discharge of her professional duties a fully qualified, refined, and educated woman, at once firm and sympathetic, may find a singularly suitable and beneficent vocation.

### CARSHALTON COTTAGE HOSPITAL.

With reference to the remarks in our issue last week under the heading of "Friction in a Cottage Hospital," we learn with pleasure that things are now going on more smoothly. The matron, Miss Mustard, who had tendered her resignation, has consented to remain, and the hon. secretary, who had also resigned, has withdrawn his resignation. The nurse who was so abruptly asked by the committee to leave has intimated that she could not under any circumstances return, but it is proposed to give the matron the assistance of two staff nurses, and at her convenience she is to take a month's holiday.

### DEATH OF THE MATRON OF VENTNOR HOSPITAL.

We regret to announce the death, at an early age, of Miss C. Stuart Cameron, matron of the Royal National Hospital for Consumption, Ventnor. It was only on April 15th that an account of an interview by our Commissioner with Miss Cameron appeared in our columns, and she was then apparently enjoying excellent health. It was, however, found necessary for her a short time since to undergo an operation at St. Thomas's Home, and, unhappily, she did not survive it. Trained at the Royal Infirmary, Dumfries, and subsequently sister at Cardiff Infirmary, night superintendent at Preston Royal Infirmary, assistant matron at Sheffield Infirmary, and assistant matron at the City of London Hospital for Diseases of the Chest, she only took up her position as matron at Ventnor last autumn. She had entered into her new work with much spirit, and had made her influence felt in the institution, in which she took the deepest interest.

### CITY OF LONDON HOSPITAL FOR DISEASES OF THE CHEST.

The annual strawberry feast given to the nurses of the City of London Hospital for Diseases of the Chest, Victoria Park, by the Chairman of the House Committee, Mr. Masterman, took place last Saturday afternoon in the grounds of the hospital. Each nurse was allowed to invite a friend, and the mingling of blue uniforms with the visitors' dresses on the well-shaded lawns made a very pretty scene. A band was in attendance, and the tennis courts were in constant use. The matron and home sister spared no pains to make the afternoon a success. They also arranged that the nurses relieved each other constantly, so that all were able to enjoy the garden party. The new nurses' home at the hospital will be opened on Wednesday, July 26, by the Duke and Duchess of Connaught.

### HUDDERSFIELD INFIRMARY.

On Thursday last week an interesting little ceremony took place at the Huddersfield Infirmary. It was the second annual presentation of the Gold Medal, which is offered yearly for competition amongst the probationer nurses, and is awarded upon the result of the examination in the theory and practice of nursing. This year all the candidates did well, none gaining less than 50 per cent. of marks. The two at the head of the list were Nurse Beckett 97 per cent., and Nurse Woodhead 95 per cent. Mrs. Tom Brooke presented the medal to Nurse Beckett, congratulating her upon her success. Both candidates at the head of the list received a book. Subsequently the members of the Board who were present and other friends had tea with the nurses in the garden, which was looking its very best.

### FATAL ACCIDENT TO AN ASYLUM MATRON.

A fatal accident occurred last week to Miss Shawyer, deputy matron of the County Asylum, Whitecroft, Isle of Wight. Miss Shawyer was cycling down the Carisbrooke County Hill in the island on a new free-wheel bicycle without her brake on. Just before she reached the bottom of the hill, having with some difficulty avoided a horse and van, she overtook a boy, and, apparently having lost all control of her machine, she ran into him without ringing her bell, and was thrown heavily off, sustaining severe fracture of the skull. She died in a couple of hours. At the inquest the medical superintendent of the asylum said that by her invariable amiability and pleasantness Miss Shawyer, who in five years rose from the position of nurse to that of deputy matron, had become universally liked, and was most reliable in the discharge of her duties.

### DISTRICT NURSES IN IRELAND.

The second annual report of Lady Dudley's scheme for the establishment of district nurses in the poorest parts of Ireland shows that there are now 11 nurses at work, the cost of each being about £100 a year. Last year the number of cases attended was 1,395, and of visits paid 17,081. These figures require to be read in the light of the fact that long journeys over rough roads in all kinds of weather are undertaken. The ministrations of the nurses are so much appreciated that the committee are overwhelmed with appeals to send more to different parts of the sister island, but they cannot respond as they would wish owing to lack of the necessary funds.

### DISTRICT NURSING IN GLASGOW.

The tenth annual meeting of the Maryhill District Nursing Association was held on Friday last. The report, which was adopted, showed that the two nurses attended 324 cases and paid 6,377 visits. An extremely satisfactory feature is that the number of subscribers has increased yearly ever since the Association was founded, and now stands at 2,532, the increase for last year being 96. Unfortunately, in spite of this, there had been a slight fall in the total income, which, in the opinion of the Committee, is accounted for by the state of trade. We congratulate the honorary secretary upon the admirable manner in which the report is drawn up, and especially upon the details which are given of the sums collected in each district in the Maryhill Ward of the City of Glasgow.

### The Nursing Outlook.

"From magnanimity, all fear above;
From nobler recompense, above applause,
Which owes to man's short outlook all its charm."

### HIGHER TRAINING AND ECONOMICS.

In our issue of the 17th ultimo we drew attention to the need for the enforcement of economy in every department of a hospital. To-day we propose to consider hospital economics from the standpoint of the higher education of nurses so as to fit them for the most responsible posts in the nursing world. At the present time Great Britain contains no educational centre which devotes itself to the preparation of certificated nurses for the higher and more responsible positions in hospital work, including those of matron and superintendent of institutions, and teacher and instructor of probationers in various departments of nursing. In the United States it was recognised some years ago that it was hopeless to expect a supply of fully qualified candidates for the higher positions of the nursing world unless or until arrangements had been made to establish an educational centre which would provide capable teachers, so that all who may have to train nurses shall first of all be themselves efficiently taught and prepared for the work.

We have called attention more than once in these columns to the class in Hospital Economics which was started by Bedford College at our suggestion. We should like to see further developments in teaching in this direction, to have them associated with the principal nurse-training schools and with those who take the keenest and most active part in the training of nurses. It is essential that the regular course of subjects should be most carefully selected, with due regard to the circumstances, that the training shall include the most advanced work and be calculated to enable the students to qualify effectually for the discharge of the responsible duties attaching to the highest nursing posts throughout the country. An entrance examination to test the average general standard of education of each candidate is essential. The entrance regulations should further require evidence that the nurse candidates have been thoroughly trained in practical nursing, as well as in special subjects. The course itself should include household chemistry, home sanitation, food values and the methods of food production, instruction in hygiene, biology, and bacteriology with practical work where necessary in suitable laboratories, education and psychology, the last two to secure that the students may be taught how to teach. The course on hospital economics must be a practical course in which instruction is given through lectures and visits of inspection to institutions. It should include hospital and training school organisation, administration, with a general outline of the chief points essential in the construction and equipment of buildings, the organisation and management of departments, wards, dispensaries, matrons' and home sisters' offices, the duties of various officers, the distribution of work and its sub-division in large institutions, the keeping of accurate records upon an approved plan, and the best way to superintend and administer large establishments. No school of hospital economics in this country can be deemed to be satisfactory which does not include amongst its teachers some of the most representative and knowledgeable of the existing matrons and superintendents of nurse-training schools, and of medical officers engaged in the instruction of probationers.

Of course the organisation of a school of higher education and advanced subjects such as we have here sketched must take time and cost money. We believe it will be found, however, having regard to the urgent necessity which many feel for such a school of hospital economics, that a few, at any rate, of the brightest and most capable hospital matrons and superintendents would give a certain amount of their time to the work of establishing such a school, and we make no doubt that adequate funds would be forthcoming if the plan agreed upon was thoroughly practical and worthy of the support of all who are keenly alive to the value and need of higher education of this kind. It would be a considerable step in advance if arrangements could be made for the students to live in or near the college, and for them to be placed under the direction of a trained nurse who has herself acquired much of the higher education it would be the duty of the school to provide.

In the United States the expenses have been met almost entirely by a small group of women, chiefly nurses, who have year after year contributed from their own earnings to pay the salary of the instructor and some of the expenses of the lectures. The scheme has been aided by the voluntary services of several of the lecturers, who have met their own expenses and cheerfully given much time and thought, which could be ill spared from their busy lives, to carry on what they believe to be a necessary and important work. The expenses of the general course have been borne by the Teachers' College, Columbia University. We are most anxious to see a similar school organised in Great Britain during next autumn. We shall be very glad to receive offers of co-operation accompanied by suggestions, and to learn the views of any of our readers who may have given time or thought to the subject. It is at least one which should commend itself to the warm sympathy of all teachers and managers of hospitals which at present train large numbers of nurses, who must be fully alive to the importance of securing for the higher posts women who have been specially trained for the work.

### Medical Electricity and Light Treatment.

By Kate Neale, Sister-in-Charge of the Actino-Therapeutic Department, Guy's Hospital.

### II.—GALVANISM AND FARADISM.

Galvanism and Faradism, the forms of electricity which will be described in this and the succeeding article, are probably more often used in treatment than any others. Those of you who are private nurses will find that the majority of cases you have to deal with will be undergoing one or both of these forms of treatment, and if you want to obtain a maximum of success you must give close attention to every detail in the method, as well as to the instructions of the doctor under whom you are working. Failure to obtain the results you want almost always means some oversight or carelessness in preparing the patient or in fitting the apparatus—a sponge has been used unmoistened, or a terminal has not been screwed up tightly. Lack of cleanliness, too, in any part of the instrument, is a fertile source of failure. I have known instances where batteries and other instruments have been returned to the makers for repairs when all that was needed was a little more careful attention to the details of arranging the appliance. Always, then, bear this point in mind: If at any treatment you fail to get the effect you want, go carefully over your instrument, especially wires and terminals, to make sure that each part is connected up in the right way. If you do this you will spare yourself (and your patient) much annoyance, to say nothing of saving the expense of having your apparatus examined by the makers.

Galvanic and Faradic currents differ from each other in so many respects that it will be simpler to describe them independently, though much that you will find under the first heading will be just as applicable to the second.

### 1. Galvanism.

Galvanism takes its name from an eighteenth-century scientist, Galvani, and consists in the application of a continuous current to the patient; such a current, for example, as we have seen is obtained by connecting the two poles, positive and negative, of a cell. But instead of joining the poles directly, the free ends of the positive and negative wires are brought into contact with two different parts of the patient's body, so that the current coming down from the battery passes through him before returning along the negative wire. As a matter of practice you do not let the bare ends of the wires touch the patient, as this would be unsatisfactory in many ways, but you join them to special endings which are termed electrodes. These will be described immediately. With such an arrangement the only sensation usually produced is a tingling feeling when the current enters and leaves the body.

*Apparatus.*

There are many forms of portable apparatus for applying galvanism, but it will be unnecessary to worry you with descriptions of more than one pattern because the instruments of different makers, although they may vary in appearance, are similar in principle, and one account will serve for all. In the electrical departments of hospitals, where there is no need to have an instrument that can be moved about, both galvanic and faradic appliances are generally arranged together on what is called a switch-board; an account of this will be found at the end of the description of Faradism.

A galvanic apparatus consists of a battery made up of 20 to 60 cells, usually of the Leclanché variety, connected to two screw terminals from which the current is led off in the usual way by wires ending in electrodes. In the form shown in fig. 1 the battery is contained inside the box, and is therefore not seen; A and B are the two screw terminals to which the current is brought from the battery underneath, the positive pole being A, which is therefore the anode (indicated by the sign +), the negative pole B, the kathode (with sign −), and it is to these two terminals that you must connect your wires. Electric wires are made of copper (you will remember we saw in Section I. that all metals conduct electric currents easily) wrapped round with either india-rubber or silk to prevent any escape of current if they happen to be touched, since neither rubber nor silk will conduct electricity. The ends of any wire you are going to

Fig. 1.—Collecting Dial.

use must be bared for about an inch, to expose the copper, and it is these ends you place in the terminals, or attach to electrodes. You will find it useful occasionally to scrape the bared ends to make them clean and bright again. To the left of A you will see a sort of dial, D, which is a contrivance for making the current stronger or weaker as occasion arises. It consists of a number of little metal knobs, each being connected with one of the cells below; E is a handle which can be moved round from one knob to another. If you turn it to lie over the first knob, the current will come from only one cell, if you shift it to the second, from two cells, and so on. Each knob has a number marked against it, and it is therefore quite simple to know what strength of current you are giving. There are two points you must be careful about in using the *Collecting Dial* as it is called. The first is, never let the handle lie half on one knob and half on another; if you neglect this you will very soon wear out your battery. Secondly, always turn the handle back to the number 0 when you have finished your treatment. If you look at the figure again you will see an arrangement marked C. This is called a *Reverser*, and is used in this way. It moves to and fro by means of a handle between the letters R and N. When it is pushed over towards N (normal) the current comes out in the ordinary way from the anode A, and returns back to the kathode B. But when you switch it across to R (reverse) the direction of the current is reversed, and the current now comes out at B (which therefore becomes the anode) and returns by A (now the kathode). This device is very useful when testing muscles, for, instead of having to undo your electrode wires and connect them up again to the opposite terminals, you merely need to switch the reverser across from N to R, and what was your anode wire is now your kathode, and *vice versâ*.

*How to Distinguish Anode from Kathode.*

If at any time when the current is flowing you are uncertain which is the anode wire and which the kathode, you can identify them by one of two simple experiments.

(i.) Place the free ends of the two wires a few inches apart in a basin of water. The kathode will immediately become covered with bubbles of gas. A few may collect round the anode, but never to so marked an extent as at the kathode.

(ii.) Wet a strip of blue litmus paper with water, and press the ends of the two wires on it. In the neighbourhood of the anode the paper will turn a bright red.

(*To be continued.*)

### Nursing the Schoolboy.

BY THE MATRON OF DENSTONE COLLEGE SANATORIUM.

*Concluded from page 244.*

A TRAINED nurse, when she undertakes the position of matron or nurse in a school sanatorium, should always bear in mind that boys do not come to school to be ill, and though she may enjoy nursing what from her point of view is "a good case," the greater credit, I think, is due to the matron or nurse who by her care and foresight helps to keep the school in good health, rather than to one who nurses a boy successfully through a dangerous illness.

The main idea to be kept in mind is that parents trust us with their nearest and dearest, and that a life lost or health impaired through carelessness can never be given back. It is very hard for a mother to part with her boy for more than three-quarters of the year; it must be one of the trials of motherhood, and is deserving of our help and sympathy. The boy, on his side, although he gains ultimately by being sent early in life to a public school, loses much that "as a little boy" would be very helpful to him; and although boys are as a rule very plucky, and too proud to wear "their hearts on their sleeve," they feel leaving home keenly, and "home-sickness" is a very real pain.

Many boys during their first week or two at school suffer in silence acutely, and it is sometimes worth while, out of school ours or on Sunday, to suggest to a boy who looks unhappy hat "he does not seem very well," and when you have him, ive him a cup of tea or a basin of bread-and-milk, and show that you take a little interest in him. The thought that mong 300 or 400 new faces one person cares for him individually cheers him up, and as a rule prevents a second attack of "home-sickness," and though he will say nothing, or very little, you will discover later, directly or indirectly, how grateful he felt and what a help the small attention was to him.

In the school in which I have the privilege of working I am allowed to invite all the new boys to tea on the first Sunday of the term, and in this way I become acquainted with every boy in the school, and they know to whom to come when they do not feel well.

INFECTIOUS DISEASES.

One of the greatest anxieties to those who are responsible for the health of the schoolboy is the danger of infectious diseases. For the first three weeks of term all in authority keep a very sharp look-out for the first symptoms of the usual infectious illnesses to which the young are susceptible. The nurse herself should never relax her vigilance until the last day of the term, as on more than one occasion in my experience boys have developed chicken-pox and mumps during the last week.

With care I am quite convinced that it is possible to check the spread of infectious diseases in schools. In this school chicken-pox has been prevented from spreading on two occasions; on one it was confined to six cases, on the second to one only. Also with mumps an outbreak has disappeared after the first two cases.

Of course, strict isolation of the patient is the first step, and although it is impossible to disinfect the whole school, the room or dormitory in which the patients have slept should be thoroughly disinfected on the day they show the first symptoms.

The boy's bedding should accompany him to the sanatorium; every bed should be stripped, and the bedclothes divided, and the mattresses stood up on end so that the disinfectant can penetrate through them. The doors, windows, and fire-place should be closed, and after conveying two or three buckets with hot cinders into the room—the number varying according to the size of the apartment—two or three ounces of pure carbolic should be poured on each, the room left quickly, and it should remain closed for at least six hours. The next step, after the given time has expired, is to light a fire, open all the windows, have the bedsteads and floor washed with a weak solution of carbolic, leaving the remaking of the beds until the last minute, so that as little as possible of the unpleasantness of the disinfectant remains to annoy the occupant.

If the patient has developed the disease early in the day the disinfecting can be done before bedtime; but if, unfortunately, the discovery is not made until evening, the boys must sleep in the dormitory that night, after the infected bedding has been removed, and the room must be purified the next day.

I do not think that it is wise to move the boys from the infected dormitory and scatter them over the building wherever there happens to be room; the next best thing, if one cannot arrest the spread of disease, is to localise it.

Of course the doctor decides the period of isolation for the patient, and it only remains for the nurse to see that his directions are faithfully and effectually carried out, and that when the patients are allowed out of doors for exercise there shall not be any direct or indirect communication with the school; also she must exercise great care that all books, games, etc., are thoroughly disinfected or destroyed, and this is especially important when the same sanatorium is used for general cases.

In our school we are fortunate not only in possessing a modern sanatorium, but also a most effectual and up-to-date disinfecting apparatus, which renders everything passed through it absolutely free from infection. I need not enter into the details of disinfecting the patient, etc. Every nurse learns this practically in hospital, and those who are untrained learn it from experience and from the many admirable books already written on the subject.

When there is infection, especially if the disease be only chicken-pox or mumps, it is a pity to make too much fuss, and alarm the school by isolating wholesale. The patient should be isolated and disinfected as far as possible, but unless there is definite evidence for individual isolation, it is better to let things go on as usual. For a whole "house" or dormitory to be absent from "form" or chapel for instance, merely creates comment, and boys often write home in such a manner that the worst conclusion is formed, and not only is harm done to the school, but a great deal of unnecessary anxiety is caused.

### Queen Victoria's Jubilee Institute for Nurses.

Her Majesty Queen Alexandra has been graciously pleased to appoint the following as Queen's Nurses, to date July 1st, 1905.

### ENGLAND.

| Name | District Training at | Serving at |
|---|---|---|
| Caroline Millward Coaling .. | Bangor .. | Minchinhampton |
| Marion Frazer Cotts .. | Bermondsey .. | Whitley |
| Minnie Matilda Tomanzie .. | „ | Colchester |
| Mary Walthew .. .. .. | Birmingham (Moseley Road) | Birmingham |
| Mary Louisa Catherine Browne | Birmingham (Newhall Street) | „ |
| Jane Priscilla Kearton .. | Blackburn .. | Blackburn |
| Emily Frances Twist Watham | „ | „ |
| Mary Barker .. .. .. | Bloomsbury .. | Measham |
| Emily Georgina Barnes .. | „ | Gloucester |
| Edith Eleanor Biggs .. .. | „ | Paignton |
| Dorothy Frances Bracewell .. | „ | Gloucester |
| Gertrude Mary Myddelton Evans | „ | Alcester |
| Louisa Clara Harding .. .. | „ | Bridgwater |
| Edith Plumbly .. .. .. | „ | Lower Sydenham |
| Mary Creighton Reid .. .. | Bolton .. | Bolton |
| Hannah Emily Abbott.. .. | Brighton .. | Burnley |
| Kate Beck .. .. .. | „ | East Hendred |
| Florence Elizabeth Knight .. | „ | Wimbledon |
| Clara Louise Naunton.. .. | „ | Brighton |
| Gertrude Alice Sears .. .. | „ | „ |
| Elizabeth Drewry .. .. | Burnley .. | Burnley |
| Elizabeth Alice Till .. .. | „ | „ |
| Edith Anne Todd .. .. | Camberwell .. | Manchester (Bradford Home) |
| Matilda Weldon.. .. .. | „ | Crook |
| Marion Braddock Berrett .. | Cardiff .. | Gillingham |
| Jessica Cato .. .. .. | „ .. | „ |
| Edith Maud Morris .. .. | „ .. | Kilburn |
| Ethel Gwenllyan Williams .. | „ .. | Bath |
| Alexandrina Grant .. .. | Carlisle .. | Waterfoot |
| Alexina Cowee .. .. .. | Chelsea .. | Kettering |
| Lizzie Ann Dow .. .. | „ .. | Cleckheaton |
| Priscilla Parker .. .. | „ .. | Darwen |
| Maude Cowell .. .. .. | East London.. | East London |
| Janet Elizabeth Mundy .. | „ .. | Bassingbourne |
| Edith Margaret Jane Bowers | Gateshead .. | Gateshead |
| Christine Stuart Craik .. | „ .. | „ |
| Edith Florence Horne.. .. | Gloucester .. | Gloucester |
| Annie Elizabeth Willings Mountain .. .. .. | „ .. | Little Berkhampstead |
| Charlotte Wray.. .. .. | „ .. | Bacup |
| Margaret Emma Brown .. | Hammersmith | Hammersmith |
| Constance Evelyn Salmon .. | Haslemere .. | Haslemere |
| Florence Cornwall .. .. | Huddersfield.. | Huddersfield |
| Katherine Amelia Dolby .. | Hull .. .. | Hull |
| Mary Bird Galloway .. .. | Liverpool (Central Home) | Liverpool |
| Jessie Bruce Kennedy .. .. | „ | |
| Eliza Wright .. .. .. | Liverpool (Central Home) | Liverpool |
| Mary Bradbury.. .. .. | (Kirkdale Road) | „ |
| Catherine Gill .. .. .. | „ | „ |
| Fanny Hill .. .. .. | Liverpool (Shaw Street) | „ |
| Louisa Elizabeth Hyland .. | „ .. | „ |
| Ethel Ward Blackler .. .. | Manchester, Harpurhey | Manchester |
| Gertrude Bayley .. .. | Manchester, Hulme | „ |
| Margargaret Frances Naylor.. | Plaistow .. | Cresswell |
| Alice Jessie Gibson .. .. | Portsmouth .. | Loughborough |
| Eliza Petty .. .. .. | „ | Portsmouth |
| Clarinda Catherine Tymms .. | „ | Colchester |
| Charlotte Barbara Stanforth | Reading .. | Reading |
| Agnes Cairns .. .. .. | St. Helen's .. | St. Helen's |
| Janet Watson .. .. .. | Salford .. | Salford |
| Julia McGrath .. .. .. | Sheffield .. | Sheffield |
| Minnie Dixon .. .. .. | Shoreditch .. | Newmarket |
| Gertrude Patty Young .. .. | „ | Totnes |
| Ethel Mary Jones .. .. | Southampton.. | Southampton |
| Mabel Winifred Shingleton .. | „ | Burgess Hill |
| Elleen Elizabeth Smith .. | „ | Southampton |
| Bertha Taylor .. .. .. | Sunderland .. | Sunderland |
| Annie Cook .. .. .. | Torquay .. | Torquay |
| Eliza Cooke .. .. .. | Walworth .. | Alderley Edge |
| Jessie Ellen Pallant .. .. | „ | Great Shelford |
| Jean Eliza Key .. .. .. | Woolton .. | Woolton |

### WALES.

| Name | District Training at | Serving at |
|---|---|---|
| Deborah Walmsley .. .. | Bangor .. | Bangor |
| Margaret Elizabeth Cole .. | Stockton - on - Tees .. | Barry |
| Elizabeth Baister Sprintall .. | Stockton - on - Tets .. | „ |
| Harriet Matilda Amelia Battye | Beaumaris .. | Beaumaris |
| Dorothy Mary Burbidge .. | Camberwell | Montgomery |
| Ellen Jane Davies .. .. | Liverpool (Overton Street) | Brymbo |
| Margaret Anne Jones .. .. | Liverpool (Shaw Street) | Ton |
| Eliza Dean .. .. .. | | Newtown |
| Kate Louise Stuart .. .. | Portsmouth .. | St. Bride's |
| Edith Isabella Townsend .. | „ | Ton |
| Frances Ellen Buckingham .. | Salford .. | Ruthin |
| Annie Phillips .. .. .. | „ | Swansea |

### SCOTLAND.

| Name | District Training at | Serving at |
|---|---|---|
| Isabella Campbell Bell.. .. | Edinburgh .. | Larkhall |
| Barbara Chesney .. .. | „ | Edinburgh |
| Roberta Temple Cooper .. | „ | „ |
| Susan Morrison Cresser .. | „ | Larkhall |
| Amy Frederika Frost .. .. | „ | Edinburgh |
| Clara Asenath Jackson .. | „ | Lochwinnoch |
| Helen Leed .. .. .. | „ | Edinburgh |
| Wilhelmina McKinnell .. | „ | „ |
| Jane Anna Maclauchlan .. | „ | „ |
| Mary Anderson Murray .. | „ | „ |
| Mary Duncan Owen .. .. | „ | „ |
| Catherine MacLean Rankin .. | „ | „ |
| Selina Leslie Stewart .. .. | „ | „ |
| Isabella Truesdale .. .. | „ | „ |
| Janet Tulloch .. .. .. | „ | „ |
| Lucy Margaret Kyle MacCulloch .. .. .. .. | Aberdeen .. | Aberdeen |
| Margaret Edgar Cochrane .. | Glasgow .. | Glasgow |
| Elsie Mackay .. .. .. | „ | „ |
| Jessie Melville .. .. .. | „ | „ |
| Barbara Jackson Wilson .. | „ | „ |

### IRELAND.

| Name | District Training at | Serving at |
|---|---|---|
| Catherine Emma Brady .. | Dublin (St. Lawrence's) | Anagary |
| Madeline Kate Cockle .. .. | „ | |
| Margaret Comerford .. .. | „ | |
| Alice Doyle .. .. .. | „ | Galway |
| Marian Loughrey .. .. | „ | Naas |
| Lucy McCotter .. .. .. | „ | Waterford |
| Isabella Monahan .. .. | „ | Oughterard |
| Charlotte Anne Baxter .. | Dublin (St. Patrick's) | Cork |
| Frances Isabel Corneille .. | „ | Dublin |
| Annie Lucy Dowling .. .. | „ | „ |
| Grace Hardy .. .. .. | „ | Enniskillen |

### To Nurses.

We invite contributions from any of our readers, and shall be glad to pay for "Notes on News from the Nursing World," or for articles describing nursing experiences at home or abroad dealing with any nursing question from an original point of view, according to length. The minimum payment is 5s. Contributions on topical subjects are specially welcome. Notices of appointments, letters, entertainments, presentations, and deaths are not paid for, but we are always glad to receive them. All rejected manuscripts are returned in due course, and all payments for manuscripts used are made as early as possible after the beginning of each quarter.

### The Nurses of Fulham Infirmary.

By OUR COMMISSIONER.

### OPENING OF THE NEW HOME.

In brilliant sunshine on the afternoon of Thursday last week Her Royal Highness Princess Christian formally opened the new Nurses' Home in the Fulham Palace Road, which has been erected by the Board of Guardians to provide accommodation for the nurses of the Fulham Infirmary. The home—which is on the other side of the street to the Workhouse and the Infirmary—is a remarkably handsome building of red and white, more suggestive of a country mansion standing in extensive grounds than of an ordinary house in a prosaic London suburb. It has a very wide frontage to the road, though a portion is built on at right angles to the rest owing to the position being a corner one.

The Princess was received by the Chairman of the Board, the Mayor and Mayoress of Fulham, the Architect, the Medical Superintendent, the Matron and others who were all presented to her, and after an inspection of the building, of which she expressed her entire approval, she proceeded through the conservatory to the daïs in the garden. Here an address was read, Miss Annie Northcroft sang "The Worker," by Gounod, the Princess declared the home open, and after a resolution of thanks, the Benediction, and the National Anthem, the ceremony came to an end.

THE NURSES' HOME, FULHAM INFIRMARY.

Owing to the large number of visitors, and the many calls upon the matron's time, it was obviously impossible to have a talk with her that day. But subsequently the occasion being appropriate for an interview about the nursing in the Infirmary I saw Miss Ballantyne on the subject. She was naturally enthusiastic about the proceedings of the day before, and the addition to the comfort of the staff.

I asked whether she herself was going to reside in the new home.

"No. I remain where I am. The Guardians have been exceedingly kind, and a room has been built for me, and they have pressed me to go and occupy it; but I am quite sure that the proper place for a matron is amongst her patients. So the assistant matron, Miss M. J. Hughes, will take charge of the Home."

"How many nurses can you accommodate in it?"

"Sixty-two; and, of course, they all have separate bed-rooms. No one sleeps at the Home except the nurses, and assistant and second assistant matrons. The servants who work it, a cook, and kitchen-maid, and four dormitory maids sleep in the Infirmary."

"Where the nurses previously slept, I suppose?"

"Yes, the day nurses slept in the Infirmary, but we were so badly in need of room that for a long time the night nurses, sixteen in number, and two night sisters, had to sleep in a hired house in Barons Court."

"Has the Home been long in course of building?"

"It was commenced soon after I came here from Shirley Warren Infirmary, and it has been very pleasant to see it gradually grow to completion."

"You were matron at Shirley Warren Infirmary, I believe?"

"Yes; and before that I was sister, night superintendent, and assistant matron at Lewisham Infirmary, having been trained at Guy's. In those days the housing of the nurses was very different to what is is now."

### THE TRAINING.

"Before I go over the Home, will you tell me something about the training here?"

"Probationers are received between the ages of 21 and 30. They have two months' trial, during which time they are given a little test paper. The way in which they answer this, together with their behaviour in the wards, and their general suitability decides us whether or not they shall be allowed to sign on for their three years."

"I suppose the questions are quite simple?"

"Oh, yes. These are the last given. (1) Describe fully your experiences during your first week as a probationer at this infirmary. (2) Describe how you would make and apply a boracic acid fomentation. (3) What is the normal temperature of the human body? What is the normal pulse rate? The first question enables us to gauge their general intelligence and their education, the other two their powers of memory and observation. Then, if all be satisfactory, they are admitted as probationers, with complete indoor uniform, and

### THE NURSES OF FULHAM INFIRMARY—*Continued.*

outdoor uniform of bonnet and cloak all provided by the Guardians. The salary commences at £10 for the first year, £16 the second, and £18 the third."

"Of course there are lectures besides instruction in the wards?"

"The first year they have lectures by the assistant medical officer in physiology and by the second assistant matron in nursing; the second and third years the medical officer and the second assistant matron lecture to them in addition to myself. They have examinations every year after the various courses and a final examination conducted by Professor Pepper of Charing Cross Hospital, before they can receive their certificate. I think it most important to have an outside examiner. If, however, in the meantime, she is considered capable of the position, a probationer may be advanced before the end of the three years to become a staff nurse at an additional salary, but the agreement to remain three years still holds good and no certificate is given until it terminates."

### Theatre Work and Off-Duty.

"How much night-nursing falls to the probationer's share?"

"After eight months probationers are generally put on to night duty, more especially if, as is the case with most of our nurses, they have had fever or children's training before coming here. They take the night work for three months, with one night off every fortnight, and they then do day and night work alternately every three months.

"You have, I know, a new theatre here. How soon do probationers help at operations?"

"Whenever the medical officer sends them, and that is as soon and as often as we can manage, quite irrespective of whether the case to be operated on belongs to their ward or to another. We hold that the more experience they get the better for them, and, after a little while, they generally like to go."

"You give maternity training here, do you not?"

"Yes, we have a maternity block, under the charge of a midwife and two trained nurses, who live and sleep there, and are quite separate from all the other nurses. One of our nurses has just passed the examination of the Central Midwives Board."

"Now as to off time?"

"All nurses have 2 to 4.30, or 7 to 10 every week day, and 10 to 1, or 6 to 10 on Sundays. In addition they have half a day once a week—from 2 to 10—and a day a month. Sisters and nurses have exactly the same, with one exception. The sisters are allowed half a Sunday every three weeks, nurses every six weeks, in addition to the other free time. As annual holiday there is 16 days' leave for nurses, three weeks for sisters."

### The New Home.

And then we crossed over to the new Home, the matron explaining as we went that, owing to a difficulty in getting the sanction of the County Council the nurses at present have to go across instead of under the street, but that will now soon be remedied. The subway is to be entirely of white glazed tiles.

The scheme of colouring (two shades of green) used in the Home is apparent at once upon reaching the entrance hall. On the right is a charming little sitting-room for the assistant matron, with a pretty dark green velvet pile carpet a fuller shade of the walls. This, the matron told me, is used all over the Home, with the exception of the bedrooms, where cork-carpet or linoleum is employed exclusively, with differently coloured rugs. On the left is the large dining hall, lighted by day by the five windows looking out on to the road, and at night by electric light, which is placed in metal chandeliers and suspended from the ceiling, the colouring being that of a green beetle's wing, containing four lights in each. The whole of the appointments are in extremely good taste, the first-rate engravings on the walls of the sitting-room, which the matron said were selected personally by the lady Guardians, being specially noteworthy.

### The Dining Hall.

Instead of one long table the Guardians have chosen small tables for the nurses, to hold four each. These will be arranged two by two down the room, but on Friday they were pushed against the wall, where they had been utilised for the refreshments the day before, thus leaving the centre of the floor free for the dancing till 10.30, with which the nurses ended the festive day. There is a fireplace each end, with pretty green tiles, though hot-water pipes also warm the house throughout, and a lift direct from the kitchen brings up the dishes straight from the fire. All meals for the nurses, even tea, are served here. Next to the dining-room is the library or quiet room, where nurses anxious to study can do so in both peace and comfort. Each of the library chairs had at the back a little green pad to match the carpet. This the matron explained was her improvised little cushion to prevent the chairs, when pushed back hastily, from damaging the dainty new wall. The glazed bookshelves of white enamel are at present unoccupied. The sitting-rooms occupy one entire end of the house, the sisters' room being in front, the nurses' at the back. They are divided by a solid partition, which can be removed upon occasions, and as the carpet and furniture are all similar the effect is very good. Each room has a "cosy corner," many sofas and lounges and a piano, but the sisters have in addition a delightful little room which is called "The Nook." It contains a writing-table and most inviting chairs for a chat. Everywhere are electric burners with soft green silk shades and long green tubes which can be carried to any table requiring additional light, and a few screens to enhance the artistic *ensemble* of the room.

### The Bedrooms.

Before going upstairs we passed through the compact little conservatory—where the assistant matron will try her horticultural powers—on to the steps leading down to the garden. Here is a full-sized tennis-lawn, the walls being well wired above so that tennis balls may not stray on to the adjoining property, the watering hose standing ready to keep the grass as green and fresh as at present. Eight bedrooms are on the ground floor, including that and the bath-room devoted to the assistant matron. Scarcely two rooms are alike in shape and design, but every one is of a good size with bedstead, wardrobe, washing-stand and dressing-table in pretty dark wood, and a movable electric light also with a green silk shade. On the half landing are three bath-rooms, and the same on the half-landing above.

The first floor is occupied largely by the sisters, to whom 15 out of the 19 rooms are assigned. The bedrooms are the same, except that a few are rather larger. No nails are allowed to be knocked in anywhere on the walls, but there are picture rails all round the room, so that knick-knacks and pictures can be hung without damage. The second floor is given over to the night nurses and sisters. Here the only addition to the rooms is a second dark green blind to be pulled down after the cream blind, which preserves the uniformity of the front of the house from outside, has been lowered. Those nurses who prefer to sleep in the light need not, of course, use their green blind. All the bedrooms have a catch on the doors of a special kind. It cannot be opened from the outside by anyone but the nurse herself with her own key, so that thefts from the rooms are impossible. In case of accident the matron and the assistant matron possess master keys which will open every door in the house.

There is ample telephonic communication with the infirmary and the workhouse, and implements for extinguishing fire are at every corner. Downstairs are a capacious kitchen and scullery, two cellars for storing boxes and trunks, a maids' mess-room, and a large hot-air room to thoroughly air all the linen as it is sent into the house; also a commodious bicycle house, with stabling for twelve machines, a separate box for each nurse's repairing tools, and a special slanting entrance from the high road, so that no dirt need be brought into any other portion of the house.

### North-Eastern Hospital for Children.

### THE NEW NURSES' HOME.

On Monday afternoon Lady Amherst of Hackney laid the foundation stone of the nurses' home which is to be erected in connection with the North-Eastern Hospital for Children, Hackney Road. Among those present were Lord Amherst and Lord and Lady William Cecil, Sir M. M. Bhownaggree, M.P., Col. De Lara Cohen, V.D., Mr. J. Goode (Executor of the Crooke Bequest), Mr. Walter Johnson, J.P., the Mayor and members of the Borough Council of Bethnal Green, Miss Phillips, and Mr. Herbert Robertson, M.P.

After Lady Amherst and Lord Amherst (President of the Hospital) had been received, and a bouquet presented to the former by one of the patients in the hospital, the company adjourned to the garden, where the ceremony took place under a gaily decorated awning. The fact that the weather was fine added to the picturesqueness of the scene. The proceedings were opened with prayer by the Rev. W. G. Morcom, M.A., Honorary Chaplain of the Hospital, and the hymn commencing

O Lord of Heav'n, and earth, and sea,
To Thee all praise and glory be was then sung by the choir of nurses, assisted by Mrs. John Bate and Miss Putney, and accompanied by the band of the East London Royal Engineers (Volunteers).

Lord William Cecil, the chairman of the hospital, then read the address to Lady Amherst, referring to the powerful support which she and the members of her family had given to tho hospital almost from its earliest years, and assuring her that her presence to mark a further step forward in its development was an especial gratification and an encouragement to all who were associated with the work of the institution. He pointed out that at present the nursing staff was accommodated in two houses, rented at £130 a year, distant nearly a mile from the hospital, and that this temporary arrangement cost the hospital £400 a year in excess of the ordinary expenditure. The executors, under the bequest of the late Mr. Arthur Ocran Cooke, having given a donation of £3,000 towards the cost of providing a home, the committee had been able to consider this important matter of a new home as a practical scheme. The cost of the building, which would contain 60 bedrooms, two sitting-rooms, six bathrooms, etc., would be about £7,300. Further sums having been added to the building fund, including a donation of £1,000 from King Edward's Hospital Fund, an amount of £2,500 for general building purposes was available, and therefore the sum in hand towards the cost was about £5,500. But in addition to the home, the committee felt that it was necessary to build a laundry, which would effect an economy of at least £400 a year, and this should, in order to save additional expense, be built before the home was completed, but as the sum required was £3,700, they were unable at present to undertake it.

Mr. Arthur G. Leighton, the architect, having handed to Lady Amherst a silver trowel with ivory handle, she proceeded to lay the stone. This ceremony having been performed with much care and precision, Mr. Leonard A. Gibbs, acting treasurer of the hospital, made a statement as to the financial position, amplifying the facts mentioned in the address.

Mr. Walter Johnson, J.P., ex-Mayor of Hackney, and member of the Committee, then expressed the thanks of the Committee to the executors of the late Mr. Arthur Ocran Cooke, for the donation of £3,000 towards the building. He said that the Committee were deeply grateful for this donation, as it had made the scheme for a home practical. They desired to thank Mr. Goode, as executor, who was present on the platform. Mr. Johnson also referred to the splendid work done by the Ladies' Association.

Sir M. M. Bhownaggree, M.P., in proposing a vote of thanks to Lady Amherst, said he did so with some personal as well as public feeling, because, with the help of Lord Reay, he had established the first nurses' home in India, in memory of a sister, and he was therefore particularly interested in the building of this new home. Mr. Herbert Robertson, M.P., seconded, and Lord Amherst replied on behalf of his wife, observing that it was a pleasure to both of them to be present on the occasion.

At the close of the proceedings Lord William Cecil announced that £500 had been promised towards the building fund by a member of the committee. The band of the East London Royal Engineers played selections before and after the ceremony. The wards of the hospital had been charmingly decorated with flowers by the nurses, and were thrown open for the visitors' inspection. The ceilings and walls in the new wards are tiled, and they all have balconies, upon which many of the little patients rest in their couches.

### The First Midwifery Examinations under the Central Midwives Board.

### BY ONE OF THE CANDIDATES.

The lower end of Savoy Street teemed with nurses early in the afternoon of June 27th. There must have been close on 400 assembled outside the east entrance of Victoria Hall for the first midwifery examination under the new rules. The clatter of tongues might have been heard in the Strand. There were nurses in black, nurses in blue, a few in green, one in grey, and half a dozen who had come in "ordinary garments" and who looked uncomfortable "and out of it" amongst all the "uniforms." "Do you feel nervous?" "Whom have you been working under?" "Can you do the 'Presentations' all right?" were the questions most frequently asked —in fact one nurse wandered from group to group collecting this or very similar information from all who would give her a minute's attention.

Evidently there were many who thought the new Board would be niggardly regarding writing materials, and arrived armed with rolls of paper; while others gave themselves away as previous failures by displaying their "own pens," having had enough of "their scratchy old things last time." As soon as the doors were opened the nurses streamed downstairs, took off their cloaks, tripped up again in indoor uniform and bonnets, and were told off by number to their various desks and rooms. A slip of paper was given to each, with the six examination questions printed thereon, and work began. The questions were as follows:—

1. Describe the shape of the female pelvis, and give the length of the principal diameters. 2. What conditions would lead you to suspect the existence of some obstruction during labour? What is a midwife's duty in such a case? 3. Describe in detail the proper management of the third stage of labour. 4. How is puerperal fever caused? State fully the precautions a midwife must take, after attending such a case, as required by the rules of the Central Midwives Board. 5. (*a*) Make a list of the important diseases which may develop during the first ten days of a child's life. (*b*) Give the signs of these diseases. 6. Under what circumstances must a midwife "decline to attend alone, and must advise that a registered medical practitioner be sent for," according to the rules of the Central Midwives Board?

As will be seen, two of these had practically to be answered word for word from the rules of the Board, and were thus easy enough for those who had studied the booklet, but "posers" for those who had not. The first question seemed to bother a few, though others laughed at its simplicity; but whether many answered it as correctly (?) as the nurse who placidly announced afterwards that she had given the "measurements of the pelvis" as about 37 to 47 inches, only the examiners know! Anyhow, nearly every one had done as much as they meant to, and given in their efforts before the allotted time had expired.

July 3rd and 4th were the days appointed for the *viva voce* part of the examination, and though there was a continuous stream of nurses in and out of the Hall, of course they did not congregate in such a mass as on the previous occasion. Taken as a whole the candidates looked more nervous on these days. Most of them sat about in groups discussing "probable" questions and advising, or being advised, as to the answers. Others there were with eyes shut and lips moving, evidently recalling all they could of whatever "book" they pinned their faith to. Those coming out from the ordeal were seized on whenever possible and examined minutely as to what they had gone through during the dreaded ten minutes. One poor nurse from one of our biggest London hospitals got much sympathy when she owned to having "muddled" each question she was asked. She accused the "second doctor at the table" of having been the cause of this, as he "kept making sarcastic remarks all the time." Her frame of mind was anything but kindly. Another was in tears—she had "lost her head," though the doctors were both of them "just as kind and helpful as they could be." Well it's all over now, except the "notifying," and one can only hope the many that pass will be happy ever after, and the few that fail will have "better luck next time."

### An Ober Ammergau Tour.

On August 11th Miss Davidson proposes to conduct a party of ladies on a tour of 25 days (divisible into 10 and 15 days). The programme includes Ober Ammergau (to see the School of the Cross). The Falls of the Rhine, Lake Constance, Bregenze, and the Bavarian Highlands and castles, Innsbruck and the Dolomites. Miss Davidson will be glad to give all particulars to Nurses and others who care to join if they will write or call at 37 Essex Street, Strand.

### Death in Our Ranks.

The death of Nurse Hilda Tovey, of Stroud Hospital (not Love), should have been announced last week.

### Where to Go.

Hospital and Home for Incurable Children, "Northcourt," College Villas Road, Hampstead.—A Pound Day and Small Sale of Work, Friday, July 21st. From 3 to 6 p.m.

### The Nurses' Bookshelf.

How to Become a Nurse. The Nursing Profession: How and Where to Train. Edited by Sir Henry Burdett, K.C.B. (London: The Scientific Press, 28 and 29 Southampton Street, Strand, W.C. Price 2s. net.)

This book serves a twofold purpose. It benefits the busy matron by saving her from innumerable and useless letters of inquiry, and it places the would-be nurse in a position to review all that the field of nursing has to offer and to select with discretion the scene of her future labours. A glance at the table of contents shows the comprehensive nature of the work. It embraces a concise and practical explanation of the usual arrangements made between the institution and the nurse; the system of training; a description of the various branches of nursing—monthly, district, mental, and special; Army, Naval, Colonial, and Indian nursing services; the administration of the Central Midwives Board and an account of the Act; poor-law infirmaries; private nursing; residential homes and clubs, provident funds, examining bodies, and associations for the benefit of nurses; and in addition a Directory, which forms the major portion of the book. The Directory is arranged on a uniform plan; the details given include official statements as to the age at which nurse candidates are accepted at each institution in the United Kingdom, particulars as to remuneration, training and promotion, accommodation and holidays, and information on all other points with which a novice could wish to acquaint herself. The book, whilst being practically essential to those who desire to train as nurses, is also instructive to those less personally interested in its contents. It is impossible when turning over its pages not to be impressed with the vast organisation of nursing in the present day. One is tempted to wish that such a guide had been in existence some twenty years ago, so that the striking changes in the conditions of the nursing service could be more fully realised. We think we are not wrong in saying that the advantages offered to nurses by one well-known training-school could then have been dismissed in a few lines of print. To-day the recitation of the curriculum and arrangements at this school occupy two entire pages of the Directory.

### Everybody's Opinion.

### PORTERESS AS MATRON.

Mr. A. W. Davis writes from the Isolation Hospital, Gamesley, Dinting, Derbyshire, under date of July 9th: It was with great surprise that I received a copy of your paper dated May 27th, containing a notice of our appointment at above, the facts of which are entirely untrue and very damaging. We have only been porter and porteress at one institution, and that we took to obtain a joint appointment, with a better one in view. There is nothing strange in what is done every week by trained people when they wish to get together. My wife is a London-trained nurse, and has held the office of superintendent and head nurse at several London institutions. She gave up a post of head nurse to join me, and I do not think that should make her unfit for the post of matron of this hospital as you infer.

[There was no intimation in the appointment that the new matron of Gamesley Infectious Hospital was a trained nurse, although we assumed that it was possible she might be; and there is, of course, no sort of discredit attaching to the position of porter or porteress. But the fact that our correspondent and his wife have only been porter and porteress in one, instead of in several institutions, does not affect our opinion that it is a disadvantage for the wife of the caretaker to be the matron of the hospital.—Editor The Hospital.]

THE GIFT OF A BICYCLE.

"B. and C." write: On August 21st, 1903, two weary midwives (Nurses B. and C.), tired out with a long difficult case, reached home in the middle of the afternoon, and while foraging round for something to eat had a visit from another nurse friend, bearing in her hand the current copy of THE HOSPITAL, containing a notice that a lady desired to give away her bicycle to a district nurse. They telegraphed at once, and Nurse C. wrote by the next post. These two had "chummed" during their training and had recently taken a cottage in a small town in the south of England, hoping to work up a midwifery practice. But Nurse B. was not strong, and the country work was growing. One deplorably wet winter Nurse C. had walked over 400 miles in her visits to only seven cases. The days passed on and we had no reply, and indeed hardly expected one. Why should we prove the lucky ones among the dozens of nurses who would apply? Then, to our joy and amazement, one Saturday morning three weeks later, a ladies' bicycle arrived! followed by a letter desiring to know how much the carriage had been, that it might be refunded, and telling us that no fewer than seven nurses had been provided with bicycles—so interested had the writer, and through her her friends, become in the letters they had received. If the advertiser could take a peep into this little home, nowhere would she find a pair of happier or busier nurses. Babies are our special work—one being always on the spot to attend to them—but a good deal of odd daily work, and occasionally a plum in the shape of a good maternity case comes our way; and since we must be at home on account of an urgent message from a hastily-arriving baby, our leisure is spent and a good deal of pleasure and profit found in a thriving poultry yard. Our domestic happiness is not marred by that questionable blessing, a maid, and Nurse B.'s clever fingers make all our uniform (except collars and cuffs), including a cloak with a removable "inside," adapting it for either summer or winter use. The cycle has been positively health-giving to Nurse B., who now rides miles to cases that she would have had to refuse if it had meant walking, and an untold boon to many patients who, from their very isolation, must have had but scant attention had it not brought a midwife within their reach and means. "Miss G." (the name bestowed upon the cycle) is in excellent condition still, and is lovingly tended and polished, etc., by her admiring owners, to whom she is almost as dear as a human being.

### Appointments.

**[No charge is made for announcements under this head, and we are always glad to receive and publish appointments. The information, to insure accuracy, should be sent from the nurses themselves, and we cannot undertake to correct official announcements which may happen to be inaccurate. It is essential that in all cases the school of training should be given.]**

BARNET INFIRMARY.—Miss Kate Mitchell has been appointed ward sister. She was trained at Shoreditch Infirmary, where she has since been staff nurse. She has also been staff nurse at the National Hospital, Bloomsbury, London.

BURY HOSPITAL AND DISPENSARY.—Miss E. Blanche Harrison has been appointed night charge nurse. She was trained at the North Riding Infirmary, Middlesbrough, and has since been staff nurse at the Park Hill Fever Hospital, Liverpool; staff nurse at Birkenhead Borough Hospital; and staff nurse and night sister at Dorset County Hospital, Dorchester.

CLAPHAM MATERNITY HOSPITAL.—Miss Ewbank has been appointed sister. She was trained at Salford Royal Hospital, Manchester, and for midwifery at Kensington Infirmary. She has since been charge nurse at Ecclesall Infirmary, Sheffield; charge nurse at the Fountain Hospital, Tooting; night sister at Wolverhampton Union Infirmary; and charge nurse at Dartford Infirmary.

GREAT YARMOUTH HOSPITAL.—Miss Mary Bemrose has been appointed matron. She was trained at Leeds Union Infirmary, and has since been nurse at Cumberland Infirmary, Carlisle, charge nurse of the surgical and accident wards and out-patients' department at Stockton and Thornaby Hospital, Stockton-on-Tees, and matron of Ebbw Vale Hospital.

GUILDFORD, GODALMING AND WOKING JOINT HOSPITAL.—Miss Katherine Emma Mayhew has been appointed charge nurse. She was trained at the Eastern Hospital, Homerton, and has since been staff nurse at Hastings Sanatorium, staff nurse at Leyton Hospital, first assistant nurse at the Fountain Hospital, Tooting, London, and assistant nurse at the Borough Isolation Hospital, Leicester.

NOBLE'S ISLE OF MAN HOSPITAL.—Miss May Caskill has been appointed charge nurse. She was trained at the Victoria Hospital, Blackpool, and has since been theatre nurse, sister and night superintendent at Newport County Hospital, Mon., and charge nurse at the City Hospital South, Liverpool.

PARK FEVER HOSPITAL, HITHER GREEN, LEWISHAM.—Miss Sadie Storey, Miss Daisy Cordingley, and Miss Rosina Wilkins have been appointed charge nurses. Miss Storey was trained at the General Infirmary, Huddersfield. Miss Cordingley was trained at the General Hospital, Perth, Western Australia, and has since been nurse matron at the Hospital, Yarloop, Australia, and head nurse at a private hospital in Bunbury. Miss Wilkins was trained at Fulham Poor-law Infirmary, and has since been assistant nurse at the Grove and North-Western Fever Hospitals, London.

PENISTONE DISTRICT ISOLATION HOSPITAL, SHEFFIELD.—Miss Florence Keene has been appointed nurse-matron. She was trained at Monsall Fever Hospital, Manchester, and at the Derbyshire Royal Infirmary, Derby, where she has since been sister of the male medical ward.

SHOREDITCH INFIRMARY.—Miss Elizabeth M. Thornewill has been appointed ward sister. She was trained at King's College Hospital, and Queen Charlotte's Hospital, London. She has since been staff nurse at King's College Hospital, and midwife under the Kingswood District Nursing Association.

THE NORTHERN INFIRMARY, INVERNESS.—Miss E. C. R. Philp has been appointed matron. She was trained at Edinburgh Royal Infirmary, and has since been assistant-matron at the Royal Berkshire Hospital, Reading.

### Novelties for Nurses.

(BY OUR SHOPPING CORRESPONDENT.)

THE "MATRON" AND THE "MAID" COSTUME CLOTH FOR NURSES.

THERE are two essential qualities in a material for a nurse's uniform. These are durability of colour and fabric. I have before me patterns of cotton cloths which fulfil these necessary conditions. The "Matron" cloth is the stouter of the two materials, which are almost similar in appearance. The colours and designs are charming, and afford suggestions for considerable variety in the choice of a uniform. The dyes are warranted fast, and the cloth does not shrink. The "Matron" cloth is 10¾d. a yard and 40 inches wide; the "Maid" cloth is 7¾d. a yard and 28 inches wide. Any draper will obtain it if it is not in his stock, and we have ascertained that, amongst others, Messrs. Owen, of Westbourne Grove, are able to supply it. It is quite worth while sending for patterns.

THE LONDON GLOVE COMPANY.

WE are asked to remind our readers that the above company is now holding a summer sale at 45 Cheapside. Bargains in fancy drapery, hosiery, and underclothing are included in the sale.

### Notes and Queries.

**REGULATIONS.**

**The Editor is always willing to answer in this column, without any fee, all reasonable questions, as soon as possible.**

**But the following rules must be carefully observed.**

1. **Every communication must be accompanied by the name and address of the writer.**
2. **The question must always bear upon nursing, directly or indirectly.**

**If an answer is required by letter a fee of half-a-crown must be enclosed with the note containing the inquiry.**

*Midwife.*

(121) I should be glad to know what hospitals would be willing to receive me as a pupil midwife? I have applied to one institution, but was refused on the ground that I had had no hospital training as a nurse.—*J. T.*

There are several institutions where you could be received as a paying pupil midwife without having been trained as a nurse, but you will have to pay a fee. For list see "How to Become a Nurse: the Nursing Profession, How and Where to Train." You could also write for advice to the Rural Midwives Association, 47 Victoria Street, London, S.W.

*Age for Training.*

(122) Will you kindly let me know where to apply for a young lady to start training for a nurse, and if she can do so at the age of 19?—*B. S.*

As a rule the large training schools will not accept as probationers women under 23, though there are a few exceptions. But even in a children's hospital the usual age is not under 21. It might be possible for her to fill up her time in some institution for chronic or convalescent cases until she is old enough to become a probationer in a good hospital. You had better advertise.

*Peptone Powder.*

(123) I should be much obliged if you would kindly tell me where peptone powder can be obtained.—*A. B.*

From any chemist.

*Probationer.*

(124) Would you be so kind as to tell me how I could obtain a post as probationer where a salary is given? I am at present a cook.—*E. M. L.*

You might write to the matron of the Middlesex Hospital, Mortimer Street, London, W.

*Massage.*

(125) I wish if possible to become a masseuse and should be grateful for any information upon the subject. I see an advertisement in your paper. Could you give me any information as to whether it would be advantageous for me to train there?—*I. P.*

You could certainly get good training at the address you mention, and could be prepared for the examination of the Incorporated Society of Trained Masseuses.

*Unfinished Training.*

(126) I am at present in a large general hospital with 280 beds, but owing to family bereavement I am unable to finish my training. I have a widowed sister with good business knowledge, thoroughly domesticated, and with furnished house. Will you kindly tell us what we could do together or separately?—*Nurse C. U. I.*

We will write to you after receiving more particulars of your circumstances.

*Queen's Nurse.*

(127) I am desirous of becoming a Queen's district nurse. Will you kindly tell me where I should write for particulars?—*L. J.*

Write to the General Superintendent, Queen Victoria Jubilee Institute for Nurses, 120 Victoria Street, S.W.

*Children's Hospital.*

(128) If a nurse has been trained in a London children's hospital for two years and received a certificate, would she be considered eligible to act as staff nurse in another children's hospital without further training?—*S. M.*

Yes.

**Handbooks for Nurses.**

| | Post Free. |
|---|---|
| "How to Become a Nurse: How and Where to Train." | 2s. 4d. |
| "Nursing: its Theory and Practice." (Lewis.) ... ... | 3s. 6d. |
| "Nurses' Pronouncing Dictionary of Medical Terms." ... | 2s. 0d. |
| "Complete Handbook of Midwifery." (Watson.) ... | 6s. 4d. |
| "Preparation for Operation in Private Houses." ... | 0s. 6d. |

Of all booksellers or of the Scientific Press, Limited, 28 & 29 Southampton Street, Strand, London, W.C.

### For Reading to the Sick.

WISDOM-WORKING SORROW.

If sorrow came not near us, and the love
Which wisdom-working sorrow best imparts,
Found never time of entrance to our hearts;
If we had won already a safe shore,
Or if our changes were already o'er,
Our pilgrim being, we might quite forget,
Our hearts but faintly on those mansions set,
Where there shall be no sorrow any more.

Therefore we will not be unwise to ask
This, nor secure exemptions from our share
Of mortal suffering, and life's drearier task.
Not this, but grace our portion so to bear
That we may rest, when grief and pain are over,
With the meek Son of our Almighty Lover.

*Archbishop Trench.*

His visitations are seasons of grace. Miss we not, for our souls, any. So shall joy spring out of sorrow, abundance out of want, comfort out of desolation, hope out of hopelessness, rest out of trouble, life out of death, from brief "afflictions" an "eternal weight of glory." God shall speak to our hearts, and our hearts shall say unto Him, "Thy Face, Lord, will I seek;" and He Himself shall be the Strength of our hearts now in this "valley of the shadow of death," Himself, "Who filleth all things," shall, "in the land of the living," and the Brightness of His Presence, "be our Portion for ever."

*E. B. P.*

Patience is the guardian of faith, the fence of love, the strength of hope, the parent of peace. Patience protects humility, keeps meekness, is the soul of long-suffering, guides gentleness, strengthens perseverance.

Patience makes the soul to be of one mind with God, and sweetens all the ills of life. It casts the light of Heaven upon them, and transforms them into goods. It makes the bitter waters sweet, the barren and dry land fruitful. Desolation it makes a loneliness with God; the parching of sickness to be the fire of His love; weakness to be His strength; wounds to be health; emptiness of all things, to have all things from Him; poverty to be true riches; His deserved punishments to be His rainbow of mercy; death to be His Life.—*E. B. P.*

Wherever, then, we may be in the course Heavenwards, morning by morning let us place before ourselves that Morning which has no evening, and purpose we to do that and that only which we shall wish we had done when we shall see it in the light of that Morning, when in the Brightness of His Presence every plea of self-love, which now clouds our eyes, shall melt away. Evening by evening, set we before us that night "wherein no man can work," resolve we by God's grace to work on the morrow, if we see it, more steadfastly the works of God. "Place daily," said a holy bishop of our own, "place daily before your eyes your end. Think most intently whose those things shall be, what they shall profit you, which shall remain after you."—*E. B. P.*

So should we live that every hour
May die as dies the natural flower,
A self-reviving thing of power,—
That every thought and every deed
May hold within itself the seed
Of future good and future mead.

*Lord Houghton.*

## Figures and Tables

**Fig. 1. f1:**
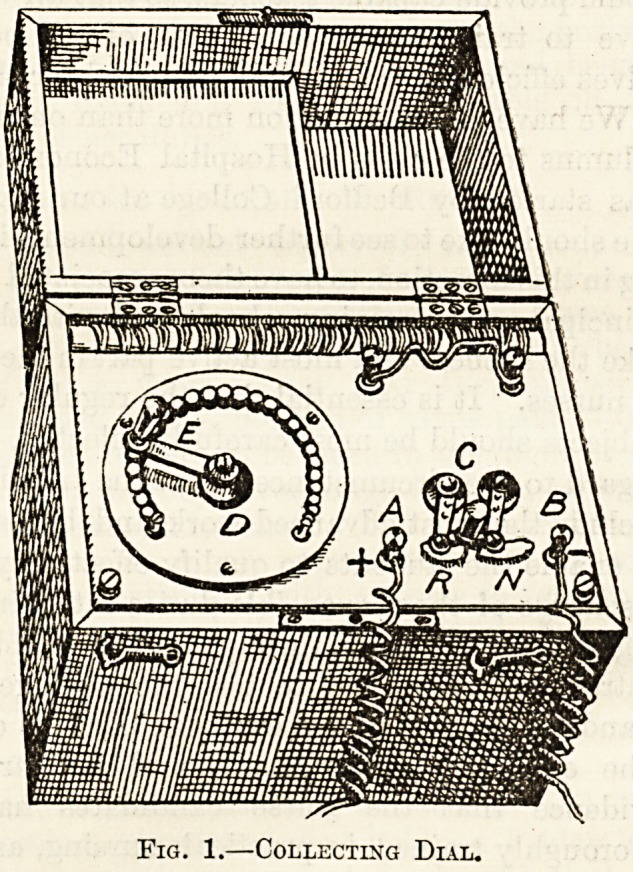


**Figure f2:**